# Long-Acting Injectable Antipsychotic Use in Children and Adolescents in Comparison to Adults

**DOI:** 10.3390/jcm14145086

**Published:** 2025-07-17

**Authors:** Iris Anja Levy, Joseph Lipton, Yoav Kohn, Alex Gizunterman

**Affiliations:** 1 Jerusalem Mental Health Center, Eitanim Psychiatric Hospital, Jerusalem 90972, Israel; joseph.l@psjer.health.gov.il (J.L.); yoav.kohen@psjer.health.gov.il (Y.K.); alex.g@psjer.health.gov.il (A.G.); 2 Hadassah School of Medicine, Hebrew University, Jerusalem 90972, Israel

**Keywords:** anti-psychotic, children, adolescents, efficacy, long-acting treatment, psychotic disorder

## Abstract

**Objective:** The aim of the study was to assess the effectiveness and safety of long-acting injectable anti-psychotic treatment (LAIA) amongst children and adolescents. Given the difficulty of performing an randomized controlled trial (RCT), we suggested comparing children and adolescents to young adults who were treated with LAIAs, and extrapolating data regarding efficacy and safety. **Method**: We compared data from medical files of adult inpatients treated with LAIAs to children and adolescent inpatients treated with LAIAs, between January 2014 and April 2021. **Results**: clinical global impression (CGI) scale score and rate of side effects (79% vs. 92%, *p*-value = 0.106) were not different between children and adolescents and young adults treated with LAIAs. There were no significant differences found between the groups in most demographic and clinical parameters such as gender distribution, legal status (voluntary or involuntary hospitalization), first hospitalizations and subsequent hospitalizations. Significant differences were found in duration of hospitalizations (144 days vs.50 days, *p*-value < 0.001), the indication for recommending LAIA treatment, diagnosis, the distribution of specific LAIAs and the rates of patients treated for side effects of anti-psychotic treatment. **Conclusions**: Results suggest that LAIA treatment may be as effective amongst children and adolescents as it is for adults. More research should be done to assess safety and efficacy of LAIA treatment in children and adolescents in the short and long term.

## 1. Introduction

The use of anti-psychotic medications in the United States amongst children younger than 12 years old is decreasing, while use among adolescents and young adults, mostly of second-generation anti-psychotic medications, is increasing [1,2]. This is understandable given the higher risk of children and adolescents for developing extrapyramidal side effects, and the relatively lower risk for those side effects occurring while using second generation anti-psychotics as opposed to first generation medications [3]. In European studies anti-psychotic treatment is less common among children and adolescents, compared with studies conducted in the United States [2].

According to the American Academy of Child and Adolescent Psychiatry, second generation anti-psychotic medications are the first line of treatment for major psychotic disorders such as schizophrenia, similar to the guidelines for treating adults [4]. In the past three decades, treatment with anti-psychotics has become increasingly common for diagnoses for which anti-psychotic medications are not formally approved [5,6,7,8,9,10,11,12,13]. In addition to their anti-psychotic effect, these medications are used as mood stabilizers and sedatives in extreme agitation or for treating conduct disorders [14,15]. The most common indications for treating children and adolescents with anti-psychotic medications are diagnoses of ADHD and conduct disorder, and they are more commonly used amongst boys [2,16]. Due to the absence of sufficient medical data as to appropriate dosage in children, the dosages being used are based on the dosages which are recommended for adults, and are usually reduced.

LAIAs have been found effective in improving adherence amongst adults and are associated with preserving remission, as well as decreasing psychotic exacerbations and hospitalizations [17,18,19,20,21]. Therefore, the total duration of untreated psychosis during the life span of a patient is predicted to be diminished. The importance of these data derives from the direct association between longer periods of untreated psychosis and worse prognosis [17,20,21]. In addition, LAIA treatment is associated with improved quality of life among adult patients [22]. Therefore, there is extensive use of LAIAs amongst adults diagnosed with schizophrenia, schizoaffective disorder and bipolar disorder [23].

In Switzerland, out of all adult patients who were hospitalized and treated with LAIAs, only 10% of patients were treated with first generation LAIAs [24]. In Canada, retrospective research examined all prescriptions for LAIAs written for adults between the years 2003–2017; the data showed that prescriptions for second generation LAIAs were written three times more frequently than for first generation LAIAs [25]. In Italy, research which included 35 medical facilities, showed that out of all adult patients who were treated with LAIAs, 70% were treated with second generation LAIAs [26]. A retrospective study in the USA examined anti-psychotic treatment in adult patients admitted between the years 2010–2016; up until 2015 there was an increasing use of second generation LAIAs as monotherapy, in comparison to use of first generation LAIAs which had a declining trend. Most of the LAIAs were of the second generation, mostly paliperidone palmitate and risperidone [27].

According to Medicaid statistics regarding 2001–2005, 75% of teenagers diagnosed with psychotic disorders and treated with anti-psychotic medications, discontinued the treatment within 18 months [28]. The well documented improved prognosis of adult patients treated with LAIAs, raises questions regarding the LAIA treatment among children and adolescents.

Data regarding the use of LAIAs among children and adolescents are sparse. Documented research is limited by small sample size, mostly open label trials, case reports or case series. Treated patients were diagnosed with bipolar disorder, schizophrenia and schizoaffective disorder. Studies regarding LAIA treatment for children and adolescents showed more use of second generation LAIAs in comparison to first generation LAIAs. The most common LAIAs in use are risperidone and paliperidone palmitate. Other LAIAs which are used but much less frequently are fluphenazine, aripiprazole, olanzapine and zuclopenthixol [6,7,10,11,29].

A study from France found that out of 167 LAIAs prescriptions written for adolescents, 86% were for second generation LAIAs, most commonly paliperidone palmitate, aripiprazole and risperidone [30].

A systematic review that evaluated studies on LAIA treatment in children and adolescents was published in March 2023 and included 13 studies [31]. Out of all the adolescents being treated with LAIAs, 18% received first generation LAIAs while all others were treated with second generation LAIAs. Overall, 53 patients were treated with risperidone, 33 patients with paliperidone, 10 patients with aripiprazole and two patients with olanzapine. 21 patients were treated solely with first generation LAIAs. In almost all of the studies clinical improvement was noted. The side effects profile varied according to the type of LAIA prescribed. Out of the studies included, one was a retrospective cohort study, focused on the use of LAI risperidone, which compared patients with adult onset and early onset schizophrenia spectrum disorders, retrospectively. The two groups were similar to each other in gender distribution and CGI-S scores. The primary outcome measured was treatment discontinuation rate, as an indicator of drug efficacy, and there was no significant difference found between the two groups [32].

It should be noted that there is a question regarding the effect of lean body mass on the absorption of LAIAs, which is especially relevant to children and adolescents [33]. Similarly, the impact of LAIAs on the occurrence of long-term side effects remains unclear.

Currently, the American Academy of Child and Adolescent Psychiatry recommends LAIAs only for adolescents who are diagnosed with schizophrenia and chronic psychotic symptoms, with poor treatment compliance [29].

Our own clinical experience at Eitanim psychiatric hospital of the Jerusalem Mental Health Center (JMHC), shows that a considerable number of admitted patients to our ward were treated with LAIAs, due to psychotic or behavioral disorders. The main indications were poor compliance and a need for urgent sedation. Those treatments were given based on clinical evidence from adult patients and in the absence of medical literature concerning the efficacy and safety among children and adolescents. The goal of this study was to provide some empirical support for the use of LAIAs in children and adolescents. As a randomized clinical trial (RCT) is difficult to perform in minors with severe psychiatric disorders, our objective was to study the outcome of the use of LAIAs in children and adolescents in comparison with young adults, all hospitalized at a psychiatric hospital. We hypothesized that effectiveness and safety will be similar in both groups, thus justifying the extrapolation to minors of evidence from RCTs in adults, supporting the use of LAIAs.

## 2. Methods

This is a retrospective study of patients who were hospitalized in the admission wards of Eitanim psychiatric hospital between the years 2014–2021. The JMHC provides services for a population of over one million people in the city of Jerusalem and its surroundings. Eitanim psychiatric hospital of the JMHC contains several admission wards for adults and one for children and adolescents. In the child and adolescent ward there is an average of 150 hospitalizations per year. The indications for hospitalization are diverse and include psychotic disorders, major mood disorders, eating disorders, PTSD and behavioral disorders. Most admissions are upon consent of the legal guardian as well as the patient (if aged 15 years old or older, per Israeli law), while approximately one third are involuntarily hospitalizations, mostly by a court order. The research sample included patients who were treated for the first time with LAIAs; minors and young adults aged 18–25.

### 2.1. Patients

1115 patients were discharged from hospitalization at the child and adolescent ward of Eitanim psychiatric hospital during the study period. 2285 men and 2511 women aged 18–25, were discharged from adult wards of the hospital at the same period. The age of 18 years was chosen as the cutoff between the two groups, as patients up to this age are admitted to the child and adolescent psychiatric ward.

A review of the medical records revealed that out of the above-mentioned patients 213 were treated with LAIAs for the first time; 167 adults and 46 children and adolescents. Out of the children and adolescents 13 were girls and 33 were boys. Among the adults there were 38 women and 129 men.

### 2.2. Study Design and Assessments

Details regarding the hospitalizations were gathered from medical records. Clinical global impression (CGI) questionnaires [34] were completed for each patient retrospectively by a psychiatrist from the research team and first author of this paper (IAL), based on the medical records on admission, during hospitalization and on discharge. CGI scales for severity of illness (CGI-S), global improvement (CGI-I) and efficacy index (CGI-E) were used. Rating of the patient’s clinical status was based on a combination of clinical experience regarding treatment of patients with identical diagnoses and collateral information.

Evaluation of treatment outcome between recurrent hospitalizations was based on medical records; if those were missing (e.g., in case there was no recurrent hospitalization or that it took place in a different facility) this measurement was not evaluated, and those patients were excluded from the statistical analysis of this measurement.

### 2.3. Statistical Methods

First, we examined the association between age group and other demographic and clinical characteristics of patients and treatment. Association between categorical variables were tested using the Pearson Chi-square test or by Fisher’s exact test, according to the small cells’ assumptions. The difference between quantitative variables of the two groups was tested by a *t*-test for independent samples or by a two sample Welch’s *t*-test, according to equality of variances by a Levene test. The second phase of data analysis included simple logistic regression models, which were used to assess the association between patients’ age group (minors vs. young adults) and other variables. Results were quantified by Odds ratio (OR), 95% confidence interval (CI) and statistical significance score (*p*-value). All hypotheses tested were two-sided and statistical significance was established by a *p*-value < 0.05. To account for multiple hypothesis testing, *p*-values were corrected using the Benjamini-Hochberg procedure controlling the false discovery rate. The data analysis was performed by R statistical software version 2022.02.0 (The R Project for Statistical Computing).

## 3. Results

As noted, 213 patients were included in this study, all of whom were hospitalized at Eitanim Psychiatric hospital between the years 2014–2021 and treated for the first time with LAIAs. 46 patients were younger than 18 years old and 167 patients were young adults, 18–25 years old. The age ranged from 9 to 25 years old, and the mean age was 20.2 years old (SD = 3 years). The majority of patients were males (76%). Table 1, Table 2, Table 3, Table 4 and Table 5 show the distribution of patient characteristics, including clinical characteristics, and outcomes of treatment by age.

There were significant differences in several categories between the two age groups. In the first phase of the comparison, significant differences were observed across several categories between the two age groups. As expected and consistent with existing literature, hospitalizations were significantly longer among children and adolescents, and the prevalence of psychotic disorders was lower in this group. The total duration of hospitalization and the duration of hospitalization after starting treatment with LAIAs were significantly longer among children and adolescents (144 days vs. 50 days, *p* < 0.001; 100 days vs. 32 days, *p* < 0.001; Table 1). Psychosis was diagnosed significantly more frequently in young adults than in children and adolescents (91% vs. 78%, *p* = 0.017; Table 1).

In the second phase, logistic regression analysis was used to examine treatment indication and discharge destination—two variables for which the observed differences were less anticipated.

There was a significant difference between the indications for treatment: among young adults the majority of patients started LAIA treatment due to lack of compliance, whereas among children and adolescents the majority of patients began LAIA treatment due to extreme agitation (95% CI: 0.17–0.69, *p* = 0.007, Table 5). Comparing the discharge destination, children and adolescents were significantly more often discharged to residential treatment centers (OR = 0.19, 95% CI:0.08–0.43, *p* = 0.004, Table 5). However, in both groups the majority of patients were discharged home (Table 1).

The specific LAIAs used were significantly different between the two age groups. Zuclopenthixol decanoate was significantly more commonly used among children and adolescents (96% vs. 43%, *p*-value < 0.001). The use of oral anti-psychotic medications and sedatives (benzodiazepines and promethazine) was significantly more common among young adults (anti-psychotics: 66% vs. 48%, *p*-value = 0.026; sedatives: 38% vs. 20%, *p*-value = 0.018). There was no significant difference in the use of mood stabilizers (Table 2).

There was no significant difference between the rates of side effects in both groups (OR = 2.96, 95% CI:0.87–10.86, *p* = 0.120; Table 5). There was a difference in the frequency of use of concomitant medication to manage antipsychotic side effects, which was significantly more common among children and adolescents (OR = 0.08, 95% CI:0.02–0.22, *p*~0; Table 5) in all three categories examined: single dose treatment, routine treatment and overall treatment (98% vs. 56%, *p* <0.001, Table 2).

Regarding the outcome assessment using the CGI questionnaire, there were no significant differences between adults and children and adolescents across all three categories: CGI-S (6 for minors vs.6.44 for young adults), CGI-I (1.76 for minors vs. 1.75 for young adults) and CGI-E (4.6 for minors vs. 4.1 for young adults, Table 4).

Young adults tended to discontinue medical treatment or change it following discharge, and the difference was close to significant in comparison to children and adolescents (OR = 3.53, 95% CI:0.95–12.66, *p* = 0.088, Table 5).

The gender distribution and hospitalization legal status were similar between the groups; in both of them approximately one third of patients were hospitalized by consent and the other two thirds were hospitalized involuntarily. The rates of first and subsequent hospitalizations were similar in both groups (Table 1 and Table 5).

## 4. Discussion

Long-acting injectable anti-psychotics are being used to treat children and adolescents despite the lack of supporting evidence for their use in the medical literature. We assume they are used mostly following lack of compliance with oral anti-psychotics. Physicians treat children and adolescents based on data regarding LAIA treatment in adults, which is well supported by clinical literature. LAIA treatment was found effective in preventing relapse in adult patients with first-episode psychosis [35]. Moreover, LAIA treatment was associated with stable improvement in quality of life in adult patients diagnosed with schizophrenia [22]. In this study, we aimed to examine children’s and adolescents’ characteristics and compare them to the characteristics of young adults (18–25 years old), all treated with LAIAs. We hypothesized that efficacy and safety of LAIA treatment would be similar in both age groups. Due to the difficulty of performing RCTs to evaluate LAIAs treatment in children and adolescents, the proof of this hypothesis would enable clinicians to extrapolate data on efficacy and safety of LAIA treatment from adults to children and adolescents.

The results of our study demonstrated that the two populations are similar in gender distribution, CGI severity scores, rates of voluntary versus involuntary hospitalizations, rates of first and subsequent hospitalizations, thus making it plausible to compare the two groups. Efficacy was determined by improvement as measured in CGI scores and safety by the rates of side effects, and both were similar and favorable in the two age groups. Children and adolescents’ condition was very much improved under LAIA treatment, as was that of young adults. Even though children and adolescents might be more vulnerable to side effects, we did not find any difference between them and young adults in this study. It might be that a longer follow up period is needed to better examine this finding.

A few parameters were significantly different between adults and children, one of them was the duration of hospitalization. Psychiatric hospitalizations of children and adolescents were found to be longer compared to adults, as was previously demonstrated [2]. This was in line with the difference which was found in the discharge destinations. Children and adolescents were more often discharged to residential treatment centers. This required preparations prior to discharge that extended the hospitalization period.

Another significant difference was the indication for LAIA treatment. Adults were mostly diagnosed with psychotic disorders and more often started the treatment due to lack of compliance with oral medications, whereas agitation was the main reason for LAIA treatment among children and adolescents. This difference between the groups complicated the comparison between them and clinical interpretation for efficacy and safety. Furthermore, the use of long-acting formulations in cases of extreme agitation might be questionable. We assumed LAIAs were used alongside with immediate release medications given orally or intramuscular, in order to reduce the use of the latter.

When examining the medications which were used, there was a significant difference between the frequency of use of medications for side effects. We have no data regarding when medications for side effects were prescribed for the first time, but we assume that those medications were used more often as prophylactic treatment in children and adolescents, since according to the medical literature this group is more sensitive to side effects [16,34]. There was also a significant difference between the rates of use of zuclopenthixol decanoate, which was more commonly used among children and adolescents. Child and adolescents were more commonly treated with first generation LAIAs [97%], mostly zuclopenthixol decanoate, whereas among adults 56% of patients were treated with first generation LAIAs, and 44% of patients were treated with second generation LAIAs. The imbalance in the type of LAIAs used between groups is a limitation in comparing the efficacy and safety between groups. It is important to note that all of the LAIAs are not formally approved for use in children and adolescents in Israel as in other countries, since their safety and efficacy in this age group have not been established. At our hospital zuclopenthixol decanoate is internally approved for the off-label use in minors, unlike second generation LAIAs. This approval is based on the long clinical experience using this long-acting formulation, in comparison to second generation long-acting formulations. Thus, it is the first choice in this age group. In both groups examined in this study, first generation LAIAs were more extensively used in comparison to data from other countries [6,7,10,11,23,24,25,26,28,29,30].

For the past two decades the use of LAIA treatment among adults was constantly rising. Among children and adolescents, the use of oral second-generation anti- psychotics was also increasing, as demonstrated in a multi-national retrospective study, which included western European countries and the USA, between the years 2005–2012 [36]. Given the lower rates of extra-pyramidal side effects using second generation anti-psychotics in comparison to first generation anti-psychotics [13,37,38,39], and given that children and adolescents are more sensitive to extra-pyramidal side effects, there is a possibility that second generation LAIA treatment should be preferred among children and adolescents. On the other hand, patients treated with second generation anti-psychotics have higher rates of metabolic syndrome [40,41], which is an important consideration when recommending anti-psychotic medication for long periods.

Among the young adults there was more extensive use of oral anti-psychotics and sedatives, which were given once or on a regular basis, combined with LAIAs. The greater staff to patient ratio in the child and adolescent ward, compared to a lower ratio in the adult wards may offer a potential explanation.

There were several limitations for the study design. First of all, the CGI assessments were performed by a single rater and in retrospective. In addition, the study lacks follow-up data after discharge, which is crucial to assess both sustained efficacy and long-term safety in pediatric population.

## 5. Conclusions and Clinical Significance

LAIAs are given to children and adolescents without having sufficient medical literature to support it.

The purpose of this study was to examine whether children and adolescents treated with LAIAs were similar in outcome to adult patients treated with LAIAs, in order to extrapolate LAIAs efficacy and safety data from adults to children and adolescents. This study was limited by a small sample size, gathering data from medical records, and CGI questioners scores assigned retrospectively. Nonetheless, the data demonstrate a similarity between patients’ characteristics of young adult and children and adolescents treated with LAIAs. In addition, LAIA treatment was as efficacious and safe for children and adolescents as it was for young adults.

This implies that clinicians are using LAIAs to treat children and adolescents, when they diagnose similar morbidity characteristics as those among adults who are treated effectively with LAIAs, even though a significant difference was found between the extent of psychotic diagnoses in children and adolescents versus adults.

It might be that this is due to physician’s rejection of traditional psychiatric classification i.e., the categorical assessment, in favor of the more modern dimensional assessment, despite lack of consensus surrounding the utility of the latter approach. The dimensional assessment suggests classifying mental disorders by integrating information of observable behavior and neuroscience-informed understanding features [42].

A significant difference between the groups was the distribution of specific LAIAs; among children and adolescents first generation LAIA Zuclopenthixol decanoate was most frequently used. Further research is needed to compare the outcomes of different second-generation LAI anti-psychotics in children and adolescents vs. adults.

The conclusion of this study was that LAIA treatment was as effective and safe among children and adolescents, as it was for adults, during hospitalizations. This is preliminary research which demonstrated the beneficial effect of LAIA treatment among children and adolescents, which can contribute to the treatment of agitated and noncompliant patients. It is important to note that LAIA treatment in children and adolescents is prescribed off-label, highlighting the need to expand age specific research and clinical guidelines. Further research is warranted to evaluate the long-term efficacy and safety among children and adolescents.

## Figures and Tables

**Table 1:** Distribution of patients’ characteristics by age.

Characteristic	Overall, *n* = 213 ^a^	Age ≤ 17, *n* = 46 ^a^	Age ≥ 18, *n* = 167 ^a^	*p*-Value ^b^
Gender				0.438
Male	162 (76%)	33 (72%)	129 (77%)	
Female	51 (24.4%)	13 (28%)	38 (23%)	
First hospitalization				0.54
No	115 (54%)	23 (50%)	92 (55%)	
Yes	98 (46%)	23 (50%)	75 (45%)	
Consent	69 (32%)	16 (35%)	53 (32%)	0.696
Duration (days)	70 (93)	144 (151)	50 (53)	<0.001
Duration after LAIA (days)	47 (73)	100 (117)	32 (44)	<0.001
Psychotic Disorder diagnosis ^c^	188 (88%)	36 (78%)	152 (91%)	0.017
Indication ^d^				0.002
No compliance	111 (54%)	16 (35%)	95 (60%)	
Severe agitation	93 (46%)	30 (65%)	63 (40%)	
Side effects				0.106
No	12 (13%)	7 (21%)	5 (8.3%)	
Yes	81 (87%)	26 (79%)	55 (92%)	
Discharge Destination ^e^				<0.001
Home	184 (86%)	31 (67%)	153 (92%)	
Residential treatment centers	29 (14%)	15 (33%)	14 (8.4%)	
Subsequent hospitalizations				0.106
No	12 (13%)	7 (21%)	5 (8.3%)	
Yes	81 (87%)	26 (79%)	55 (92%)	
Medication discontinuation ^d,f^				0.055
No	11 (7.7%)	5 (17%)	6 (5.4%)	
Yes	131 (92%)	25 (83%)	106 (95%)	

^a^ Mean (standard deviation); *n* (%); ^b^ Welch Two Sample *t*-test; Pearson’s Chi-squared test; Fisher’s exact test; ^c^ Schizophrenia, schizoaffective disorder, acute schizophrenia like psychotic disorder, acute transient psychotic disorder, Psychosis NOS, acute polymorphic psychotic disorders, bipolar affective disorder with psychotic symptoms, mania with psychotic symptoms, multiple drug use-psychotic disorder, cannabinoids-psychotic disorder-schizophrenia like; ^d^ Reference = Non-compliance; ^e^ Reference = home; ^f^ Patients with documented recurrent hospitalizations.

**Table 2:** Medications distribution by age.

Characteristic	Overall, *n* = 213 ^1^	Age ≤ 17, *n* = 46 ^1^	Age ≥ 18, *n* = 167 ^1^	*p*-Value ^2^
LAIA (Zuclopenthixol decanoate)	116 (54%)	44 (96%)	72 (43%)	<0.001
Oral antipsychotics	132 (62%)	22 (48%)	110 (66%)	0.026
Sedatives	73 (34%)	9 (20%)	64 (38%)	0.018
Mood stabilizers	79 (37%)	19 (41%)	60 (36%)	0.504
Concomitant medications for side effects-PRN use	64 (30%)	31 (67%)	33 (20%)	<0.001
Concomitant medications for side effects-regular use	131 (62%)	43 (93%)	88 (53%)	<0.001
Concomitant medications for side effects-PRN and regular use	139 (65%)	45 (98%)	94 (56%)	<0.001

^1^ *n* (%); Mean (SD); ^2^ Pearson’s Chi-squared test; Welch Two Sample *t*-test.

**Table 3:** LAIA used in both groups.

	Risperidone	Aripiprazole	Fluphenazine Dec.	Paliperidone Pal.	Haloperidol Dec.	Zuclopenthixol Dec.
Girls	0%	0%	0%	0%	0%	100%
Boys	0%	0%	0%	3.03%	3.03%	93.94%
Children & adolescents, overall	0	0	0	2.17%	2.17%	95.65%
Men	5.22%	8.21%	6.72%	29.10%	8.21%	42.54%
Women	2.63%	7.89%	2.63%	36.84%	10.53%	39.47%
Adults, overall	4.65%	8.14%	5.81%	30.81%	8.72%	41.86%

**Table 4:** CGI scores distribution by age.

Characteristic	Overall, *n* = 213 ^1^	Age ≤ 17, *n* = 46 ^1^	Age ≥ 18, *n* = 167 ^1^	*p*-Value ^2^
Severity of illness (CGI-S)	6.35 (1.26)	6.00 (1.48)	6.44 (1.18)	0.065
Global improvement (CGI-I)	1.75 (1.00)	1.76 (1.04)	1.75 (1.00)	0.943
Efficacy index (CGI-E)	4.2 (4.0)	4.6 (4.0)	4.1 (4.0)	0.439

^1^ *n* (%); Mean (SD); ^2^ Pearson’s Chi-squared test; Welch Two Sample *t*-test.

**Table 5:** Simple logistic regression analyses of the association between patient age and clinical characteristics.

Outcome	Event N	OR ^1^	95% CI ^1^	*p*-Value
First hospitalization	98	0.82	0.42, 1.57	0.630
Indication ^a^	93	0.35	0.17, 0.69	0.007
Side effects	81	2.96	0.87, 10.86	0.120
Concomitant medications for side effects	131	0.08	0.02, 0.22	0.000
Discharge Destination ^b^	29	0.19	0.08, 0.43	0.004
Subsequent hospitalizations	81	2.96	0.87, 10.86	0.934
Medication discontinuation	131	3.53	0.95, 12.66	0.088

^1^ OR = Odds Ratio, CI = Confidence Interval; ^a^ Reference = Non-compliance; ^b^ Reference = home.

## Data Availability

Due to restrictions on accessing government databases, the datasets generated for this study can be obtained from the corresponding author upon request.

## References

[1] Olfson M., King M., Schoenbaum M. (2015). Treatment of young people with antipsychotic medications in the United States. JAMA Psychiatry.

[2] Scott B.P., Waqar W., Lauren B. (2012). A Review of Pharmacoepidemiologic Studies of Antipsychotic Use in Children and Adolescents. Can. J. Psychiatry.

[3] Christoph U., Correll C.U. (2008). Antipsychotic use in children and adolescents: Minimizing adverse effects to maximize outcomes. J. Am. Acad. Child Adolesc. Psychiatry.

[4] McClellan J., Stock S. (2013). Practice parameter for the assessment and treatment of children and adolescents with schizophrenia. J. Am. Acad. Child Adolesc. Psychiatry (AACAP).

[5] Olfson M., Blanco C., Liu L., Moreno C., Laje G. (2006). National trends in the outpatient treatment of children and adolescents with antipsychotic drugs. Arch. Gen. Psychiatry.

[6] Boarati M.A., Wang Y.P., Ferreira-Maia A.P., Cavalcanti A.R.S., Fu-I L. (2013). Six month open lable follow up of risperidone long acting injection use in pediatric bipolar disorder. Prime Care Companion CNS Disord..

[7] Fu-I L., Boarati M.A., Stravogiannis A., Wang Y.-P. (2009). Use of risperidone long-acting injection to support treatment adherence and mood stabilization in pediatric bipolar patients: A case series. J. Clin. Psychiatry.

[8] Wisniewski A. (2011). New treatment option in resistant schizophrenia in adolescence-case study. Eur. Psichiatr..

[9] Eremis S., Bildik T., Tamar M., Gockay G., Karasoy H., Ercan E.S. (2007). Zuclopenthixol induced neuroleptic malignant syndrome in an adolescent girl. Clin. Toxicol..

[10] Pope S., Zaraa S.G. (2016). Efficacy of long-acting injectable antipsychotics in adolescents. J. Child Adolesc. Psychopharmacol..

[11] Lytle S., McVoy M., Sajatovic M. (2017). Long-acting injectable antipsychotics in children and adolescents. J. Child Adolesc. Psychopharmacol..

[12] Fortea A., Ilzarbe D., Espinosa L., Solerdelcoll M., de Castro C., Oriolo G., Sugrayes G., Baeza I. (2018). Long acting injectabale atypical antipsychotic use in adolescents: An observational study. J. Child Adolesc. Psychopharmacol..

[13] Vitiello B., Correll C., van Zwieten-Boot B., Zuddas A., Parellada M., Arango C. (2009). Antipsychotics in children and adolescents: Increasing use, evidence for efficacy and safety concerns. Eur. Neuropsychopharmacol..

[14] Correl C.U., Kratochvil C.J., March J.S. (2011). Developments in pediatric psychopharmacology: Focus on stimulants, antidepressants and antipsychotics. J. Clin. Psychiatry.

[15] Olfson M., Blanco C., Liu S.-M., Wang S., Corell C.U. (2012). National trends in the office-based treatment of children, adolescents, and adults with antipsychotics. Arch. Gen. Psychiatry.

[16] Pringsheim T., Lam D., Ching H., Patten S. (2011). Metabolic and neurological complications of second-generation antipsychotic use in children: A systematic review and meta-analysis of randomized controlled trials. Drug Saf..

[17] Kane J.M., Garcia-Ribera C. (2009). Clinical guideline recommendations for antipsychotic long-acting injections. Br. J. Psychiatry.

[18] Manchanda R., Chue P., Malla A., Tibbo P., Roy M.-A., Williams R., Iyer S., Lutgens D., Banks N. (2013). Long acting injectable antipsychotics: Evidence of effectiveness and use. Can. J. Psychiatry.

[19] Kaplan G., Casoy J., Zummo J. (2013). Impact of long acting injectableantipsychotics on medication adherence and clinical, functional, and economic outcomes of schizophrenia. Patient Prefer Adherence.

[20] Kishimoto T., Nitta M., Borenstein M., Kane J.M., Correll C. (2013). Long acting injectibale versus oral antipsychotics for relapse prevention in schizophrenia: A meta-analysis of randomized trials. J. Clin. Psychiatry.

[21] Mundaya J., Greeneb M., Chang E., Hartry A., Yan T., Broder M.S. (2019). Early initiation of long-acting injectable antipsychotic treatment is associated with lower hospitalization rates and healthcare costs in patients with schizophrenia: Real-world evidence from US claims data. Curr. Med. Res. Opin..

[22] Sampogna G., Di Vincenzo M., Giuliani L., Menculini G., Mancuso E., Arsenio E., Cipolla S., Rocca B.D., Martiadis V., Signorelli M.S. (2023). A systematic review on the effectiveness of antipsychotic drugs on the quality of life of patients with schizophrenia. Brain Sci..

[23] Citrome L. (2017). Long-acting injectable antipsychotics update: Lengthening the dosing interval and expanding the diagnostic indications. Expert. Rev. Neurother..

[24] Reymann S., Schoretsanitis G., Egger S.T., Mohonko M.K., Vetter S., Homan P., Seifritz E., Burrer A. (2022). Use of long-acting injectable antipsychotics in inpatients with schizophrenia spectrum disorder in an academic psychiatric hospital in Switzerland. J. Pers. Med..

[25] Roy M.-A. (2020). The evolution of long-acting antipsychotics in Quebec between 2003 and 2017. Can. J. Psychiatry.

[26] Ostuzzi G., Mazzi M.A., Terlizzi S., Bertolini F., Aguglia A., Bartoli F., Bortolaso P., Callegari C., Caroleo M., Carra G. (2018). Factors associated with first versus second generation long-acting antipsychotics prescribed under ordinary clinical practice in Italy. PLoS ONE.

[27] Liu Y., Patterson M.E., Sahil S., Stoner S.C. (2023). Inpatient prescribing patterns of long-acting injectiables and their oral or short-acting injectiable equivalent formulations. Front. Pharmacol..

[28] Correll C.U., Penzner J.B., Parikh U.H., Mughal T., Javed T., Carbon M., Malhotra A.K. (2006). Recognizing and monitoring adverse events of second-generation antipsychotics in children and adolescents. Child. Adolesc. Psychiatr. Clin. N. Am..

[29] Penfold R.B., Stewart C., Hunkeler E.M., Madden J.M., Cummings J., Owen-Smith A.A., Rossom R.C., Lu C., Lynch F.L., Waitzfelder B.E. (2013). Use of antipsychotic medications in pediatric population: What do the data say?. Curr. Psychiatry Rep..

[30] Benarousa X., Lahayea H., Cottin G., de la Riviere S.G., Guile J.-M., Speranza M., Bonnot O., Cohen D. (2022). Trends in the use of long-acting injectable antipsychotics in children and adolescents in France between 2014 and 2018. Schizophr. Res..

[31] Baeza I., Fortea A., Ilzarbe D., Sugranyes G. (2023). What role for long-acting injectable antipsychotics in managing schizophrenia spectrum disorders in children and adolescents? A systematic Review. Pediatr. Drugs.

[32] Suzuki H., Hibino H., Inoue Y., Takaya A. (2017). Treatment retention of risperidone long-acting injection in patients with early-onset schizophrenia in Japan. Asian J. Psychiatry.

[33] Fernandez E., Perez R., Hernandez A., Tejada P., Arteta M., Ramos J. (2011). Factors and mechanisms for pharmacokinetic differences between pediatric population and adults. Pharmaceutics.

[34] Guy W. (1976). Clinical Global Impressions.

[35] Besana F., Civardi S.C., Mazzoni F., Miacca G.C., Arienti V., Rocchetti M., Politi P., Martiadis V., Brondino N., Olivola M. (2024). Predictors of readmission in young adults with first-episode psychosis: A multicentric retrospective study with a 12-month follow up. Clin. Pract..

[36] Baruch Y., Kotler M., Lerner Y., Bentov J., Strous R.D. (2005). Psychiatric admission hospitalization in Israel: An epidemiologic study of where we stand today where we are going. IMAJ.

[37] Kalverdijk L.J., Bachmann C.J., Aagaard L., Burcu M., Glaeske G., Hoffmann F., Peterson I., Schuiling-Veninga C.C.M., Wijlaars L.P., Zito J.M. (2017). A multi-national comparison of anti-psychotic drug use in children and adolescents, 2005–2012. Child Adolesc. Psychiatry Ment. Health.

[38] Varimo E., Saastamoinen L.K., Ratto H., Mogk H., Aronen E.T. (2020). New users of antipsychotics among children and adolescents in 2008-2017: A nationwide register study. Sec. Child Adolesc. Psychiatry.

[39] Radojcic M.R., Pierce M., Hope H., Senior M., Taxiarchi V., Trefan L., Swift E., Abel K.M. (2023). Trends in antipsychotic prescribing to children and adolescents in England: Cohort study using 2000–2019 primary care data. Lancet Psychiatry.

[40] Rojo L.E., Gaspar P.A., Silva H., Risco L., Arena P., Cubillos-Robles K., Jara B. (2015). Metabolic syndrome and obesity among users of second-generation antipsychotics: A global challenge for modern psychopharmacology. Pharmacol. Res..

[41] Panagiotopoulos C., Ronsley R., Kuzeljevic B., Davidson J. (2012). Waist circumference is a sensitive screening tool for assessment of metabolic syndrome risk in children treated with second-generation antipsychotics. Can. J. Psychiatry.

[42] Cuthbert B.N., Insel T.R. (2013). Toward the future of psychiatric diagnosis: The seven pillars of RDoC. BMC Med..

